# Effects of *Lactobacillus plantarum* 15-1 and fructooligosaccharides on the response of broilers to pathogenic *Escherichia coli* O78 challenge

**DOI:** 10.1371/journal.pone.0212079

**Published:** 2019-06-13

**Authors:** Sujuan Ding, Yongwei Wang, Wenxin Yan, Aike Li, Hongmei Jiang, Jun Fang

**Affiliations:** 1 College of Bioscience and Biotechnology, Hunan Agricultural University, Changsha, Hunan, China; 2 Academy of National Food and Strategic Reserves Administration, Beijing, China; 3 Key Laboratory of Agro-ecological Processes in Subtropical Region, Institute of Subtropical Agriculture, Chinese Academy of Sciences, Changsha, Hunan, China; University of Connecticut, UNITED STATES

## Abstract

One-day-old broilers were randomly allocated to five treatment groups: basal diet and orally administered sterile saline (negative control, n-control); basal diet challenged with *E*. *coli* O78 (positive control, p-control); basal diet supplemented with 1×10^8^ CFU/kg *L*. *plantarum* 15–1 and challenged with *E*. *coli* O78 (LP); basal diet supplemented with 5 g/kg fructooligosaccharides (FOS) and challenged with *E*. *coli* O78 (FOS); and basal diet supplemented with both *L*. *plantarum* 15–1 and FOS and challenged with *E*. *coli* O78 (LP+FOS). The broilers in the LP, FOS, and LP+FOS groups displayed a decrease of crypt depth at day 14 compared with the control groups. Furthermore, at days 14 and 21, the broilers in the LP group exhibited reduced serum levels of diamine oxidase (DAO) compared with the p-control group (*p*<0.05), and the broilers in the LP+FOS group showed increased serum concentrations of IgA and IgG relative to both control groups and decreased DAO levels compared with the p-control group (*p*<0.05). Moreover, the LP group displayed higher levels of acetic acid and total short-chain fatty acids (SCFAs) compared with the p-control group at day 14 (*p*<0.05), and the FOS group showed higher levels of valeric acid and total SCFAs at day 21 (*p*<0.05). The LP+FOS group also displayed a higher level of butyric acid at day 14 (*p*<0.05). In conclusion, dietary supplementation with FOS improved the growth performance, while supplementation with *L*. *plantarum* 15–1 and FOS improved intestinal health by increasing the levels of SCFAs and mitigating the damage caused by *E*. *coli* O78, thus preventing intestinal damage and enhancing the immune response.

## Introduction

*Escherichia coli*-induced diarrhea has become a global public health problem in both developed and developing countries. At present, the prevention and treatment of this disease is predominantly based on vaccines and drugs. Dietary intervention has also become an important approach [1]. In addition, certain *E*. *coli* strains produce the enterotoxin responsible for colibacillosis, which is a major problem in poultry production [2, 3]. In particular, *E*. *coli* serotypes O1, O2, O78, O15, and O55 have been associated with colibacillosis in chickens [4, 5]. Among these serotypes, O78 is often associated with *E*. *coli* strains causing avian septicemia and was previously demonstrated to contain colonization factors CFA/I [6], which may undermine the immune function to predispose host animals to colonization by the pathogens, representing a threat to health and food safety. Although antibiotic therapy is effective against colibacillosis, the use of antibiotics in poultry is increasingly being limited by restrictions and bans [7]. Possible candidates to replace antibiotics include prebiotics and probiotics, which can prevent and control colibacillosis and thus protect livestock animals. Short-chain fatty acids (SCFAs) produced by the intestinal microbiota are one of the important determinants of the interaction between intestinal microorganisms and pathogenic bacteria [8]. A previous study demonstrated that dietary supplementation with lactulose improved the body weight gain and feed conversion efficiency of 21-day-old broilers but had no effect on the growth performance of 42-day-old broilers. Furthermore, lactulose treatment increased the number of colonies of *Lactobacillus* in the cecum and the levels of acetic acid, propionic acid, butyric acid, and total SCFAs in the cecum contents of 7-day-old and 42-day-old broilers [9].

Probiotics are defined as live microbial feed supplements that exert a positive influence on the host animal by improving the intestinal microecology [10]. Probiotics help maintain a healthy intestinal microflora and stimulate the immune response of the host animal to suppress the pathogenic microbiota of the gut [11]. An increasing number of well-characterized probiotic strains have been investigated to inhibit pathogenic bacteria and thus maintain a healthy avian intestinal microbiota. In particular, numerous studies have examined the influence of feeding *Lactobacillus* spp. to broilers on immune function, performance, and pathogen shedding. For example, in vitro experiments using human intestinal Caco-2/TC7 cells and intestinal explants demonstrated that lactobacilli inhibited the TLR4 inflammatory signaling induced by enterotoxigenic *E*. *coli* via modulating inflammation and the involvement of TLR2 [12]. *Lactobacillus plantarum* was also found to inhibit the growth of *E*. *coli* O157:H7 in vitro [13] and improve the growth performance, reduce the number of *Enterobacteriaceae*, and increase the *Lactobacillus* population, small intestinal villus height, and fecal volatile fatty acid concentration in broilers [14].

Prebiotics are indigestible foods or feed ingredients that positively affect the host by selectively stimulating the growth and activity of one or a limited number of bacteria in the colon [15]. Common prebiotics include fructooligosaccharides (FOS), inulin, galactooligosaccharides (GOS), transgalactooligosaccharides (TOS), and lactulose. The intake of prebiotics can regulate the intestinal microbiota by increasing the population of particular probiotic bacteria, such as *Lactobacillus* and *Bifidobacterium* [16], or competing with pathogenic bacteria for attachment sites, thereby reducing the number of pathogenic bacteria in the intestinal tract [17]. Kim et al. investigated the influence of FOS on the growth performance and immune response in broiler chickens [18]. The results revealed that dietary supplementation with 0.25% FOS had a comparable effect to avilamycin, reducing the population of *E*. *coli* and increasing the population of lactobacilli. The aim of this study was to investigate whether dietary supplementation with *L*. *plantarum* 15–1 and FOS alone or in combination reduces the negative effect on the intestinal morphology and the decline of the immune response induced by *E*. *coli* O78.

## Materials and methods

### Broilers, diets, and experimental design

All animal procedures were approved by the Animal Ethics Committee of the Academy of National Food and Strategic Reserves Administration, Beijing, China (20170052), and performed according to the guidelines recommended in the Guide for the Laboratory Animal Ethical Commission of National Food and Strategic Reserves Administration. In this study, 150 one-day-old male Arbor Acres (AA) broiler chickens with an average body weight of 46.38 ± 0.13 g were used. The broilers were obtained from a commercial hatchery (Huadu Broiler Farms, Beijing, China) and randomly allocated to one of five treatments (five broilers per pen across six pens). The negative control (n-control) broilers were intragastrically administered with sterile saline solution and separated with the rest broiler which challenged with *E*. *coli* O78. The broilers were kept in cages with a wire mesh floor and a density of 550 cm^2^/broiler. Room temperature was set to 32±2°C for the first week and gradually reduced to 24°C by the end of the third week.

The diets fed to the five groups were as follows: 1) the n-control and p-control groups were fed a basal diet without any additives; 2) the LP group was fed a diet supplemented with *L*. *plantarum* 15–1 (1×10^8^ CFU/kg of feed); 3) the FOS group was fed a diet supplemented with FOS (5 g/kg of feed); and 4) the LP+FOS group was fed a diet supplemented with both *L*. *plantarum* 15–1 and FOS (1×10^8^ CFU/kg and 5 g/kg of feed, respectively). Powdered *L*. *plantarum* 15–1 was added to the basal diet to a final colony count of 1×10^8^ CFU/kg of feed. FOS was purchased from BaoLing Bao Biology (Shandong, China, purity>95%). The components of the basal diet are summarized in Table 1. The basal diet was based on the Chinese Feeding Standard for chickens [19], was free of antibiotics, and met the nutritional requirements for starter feed (1–21 days) for chickens.

**Table 1:** Composition of basal diet.

Item	Content
Ingredient (%)	
Corn	55.75
Soybean meal	36.75
Soybean oil	2.96
Calcium phosphate	1.86
Limestone	1.2
Sodium chloride	0.35
Lysine	0.309
Solid methionine	0.287
Threonine	0.05
Choline chloride(50%)	0.26
Minerals premix^1^	0.2
Vitamin premix^2^	0.02
Total	100
Calculated nutrient composition	
ME, kcal/kg	3200
Crude protein %	22.16
Ca %	1.07
Available P %	0.68
Lys %	1.32
Methionine	0.48

^1^The vitamin premix provided the following per kilogram of diet: vitamin A, 9,500 IU; vitamin D_3_, 62.50 μg; vitamin K_3_, 2.65 mg; vitamin B_12_, 0.025 mg; vitamin B_2_, 6 mg; vitamin E, 30 IU; biotin, 0.0325 mg; folic acid, 1.25 mg; pantothenic acid, 12 mg; nicotinic acid, 50 mg.

^2^The mineral premix provided the following per kilogram of diet: Cu, 8 mg; Zn, 75 mg; Fe, 80 mg; Mn, 100 mg; Se, 0.15 mg; I, 0.35 mg.

### Bacterial preparation and oral challenge

The strain of *L*. *plantarum* 15–1 used in this study was obtained from the Academy of National Food and Strategic Reserves Administration, Beijing, China. *L*. *plantarum* 15–1 was added to the basal diet as a freeze-dried powder to a final concentration of 1×10^8^ CFU/kg. The number of colonies in the freeze-dried powder was determined by counting the number of colonies grown on plates. Briefly, 10 g of freeze-dried powder was added to 90 mL of sterile water and mixed thoroughly, the resulting mixture was diluted 1:10, and 100 μL aliquots of the dilution were plated evenly onto MRS agar (De Man, Rogosa, Sharpe). The plates were then incubated at 37°C for 10–12 h and the colonies on the plates were counted. The number of colony-forming units per gram was calculated based on the sample dilution.

*E*. *coli* O78 was obtained from the College of Animal Science and Technology, China Agricultural University, Beijing, China. The serotype of *E*. *coli* O78 was confirmed by the China Institute of Veterinary Drug Control, Beijing, China. The strain was aerobically cultured in Luria–Bertani (LB) broth for 18–24 h at 37°C with shaking at 160 rpm. A gradient dilution series (1:100) was plated evenly on LB solid media in sterile plates under sterile conditions. The plates were incubated at 35–37°C for 18–24 h and the colonies of *E*. *coli* O78 were counted [20]. The concentration after overnight culture was 3×10^8^ CFU/mL. After performing a 1:3 dilution, 1×10^8^ CFU of *E*. *coli* O78 was orally administered to the back of the oral cavity of each challenged chicken at seven days old using a sterile syringe once a day for three days.

### Growth performance

Broiler chickens were casually selected and sacrificed at days 14 and 21 after fasting for 12 h, and the body weight and feed intake of each broiler were recorded on a cage-by-cage basis. The average daily gain (ADG) and average daily feed intake (ADFI) during days 1–14, 14–21, and 1–21 and the bursal index at days 14 and 21 were calculated. The mortality was calculated over the course of the experiment.

### Sample collection

At days 14 and 21, blood samples were collected from the wing veins after fasting for 7–8 h. For each treatment, six broilers were randomly chosen from each cage and slaughtered by jugular bleeding. The bursa of Fabricius above the cloaca was weighed. A 1-cm section was cut from the center of the jejunum and fixed with formaldehyde solution for morphological examination. The cecal content was collected aseptically in sterile plastic tubes, quickly frozen in liquid nitrogen, and stored at −80°C until use.

### Jejunal morphology

The jejunal tissue samples were gently rinsed with 0.9% NaCl and then fixed in 10% buffered formalin. After fixation, the samples were embedded in paraffin, cut into 2–5 μm slices, mounted on slides, and stained using hematoxylin and eosin [21]. Complete intestinal villi were selected and the villus height and crypt depth were measured. The villus height was measured from the villus basal lamina to the villus apex, and the crypt depth was measured between the base (which is by the villus height end) and the crypt/villus transition zone [22].

### IgA, IgG, and Diamine Oxidase (DAO)

Blood samples were collected from the wing vein for the quantification of IgA, IgG, and DAO. After serum separation and centrifugation at 10000 ×*g* for 4 min, the samples were stored at −20°C. The serum concentrations of IgA, IgG, and DAO were determined by enzyme-linked immunosorbent assay (ELISA) using the Standard Chicken Kit (Nanjing Jiancheng Institute of Biological Engineering, Nanjing, China) following the manufacturer’s instructions. Briefly, the standard substance and samples were diluted to 100 μL and added into the wells and the plate was incubated at 37°C for 1 h. Next, the plate was washed three times. Next, the biotin–antibody conjugate was added into each well, the plate was incubated at 37°C for 60 min then washed three times, anti-chicken horseradish peroxidase (HRPO) was added into the wells, and the plate was incubated at 37°C for 30 min. 3,3ʹ,5,5ʹ-Tetramethylbenzidine substrate solution was added into every well and incubated at 37°C for no more than 30 min. The product concentration was then measured spectrophotometrically at 450 nm. The regression equation of the standard curve was determined from the standard concentrations and OD values, and the OD values measured for the samples were substituted into this equation to calculate the sample concentrations. All of the measurements were performed under the same conditions to minimize inter-assay variation.

### Cecal short-chain fatty acids

The SCFA concentrations were determined using the method of Schäfer [23] with some modifications. Frozen cecal digesta samples were thawed at 4°C and diluted five-fold with double-distilled water in sterile screw-cap tubes, homogenized, and centrifuged (Centrifuge 5810R, Eppendorf, Hamburg, Germany) at 10000 rpm for 10 min. 2-Ethylbutyric acid (17.01 mmol/L) was used as an internal standard. The concentrations of acetate, propionate, butyrate, valerate, isovalerate, and isobutyrate in the samples were determined using a gas chromatography (GC) system (7890B, Agilent) equipped with a flame ionization detector (FID) and a DB-FF column (30 m × 0.25 mm, 0.5 μm particle diameter, Agilent Technologies, USA). Nitrogen was supplied at a flow rate of 40 mL/min as a carrier gas. The initial oven temperature was 80°C, which was maintained for 0.5 min, and the temperature was then increased to 130°C at 5°C/min and held for 2 min, then increased to 240°C at 20°C/min and held for 1 min. The temperatures of the FID and injection port were 270 and 200°C, respectively. The flow rates of hydrogen and air as the fuel gas and oxidant gas were 40 and 450 mL/min, respectively. The GC analysis was performed using an injection volume of 1 μL and a detection time of 19 min per sample. The SCFA concentration was calculated by multiplying the raw data by the dilution factor.

## Statistical analysis

Statistical analyses were performed using SPSS for Windows. Data were analyzed using multivariate one-way ANOVA for the following parameters: ADG, ADFI, bursal index, jejunal villus height, crypt depth, immune response, and SCFA concentrations were statistical analysis, n = 6. Differences in the effects of *L*. *plantarum* 15–1 and FOS were analyzed using single degree of freedom contrast statements comparing the broilers challenged with *E*. *coli* O78 (p-control) with the unchallenged group (n-control) from 0 to 21 d. Differences were considered significant at *p<*0.05.

## Results

### Growth performance and survival

**Fig 1:**
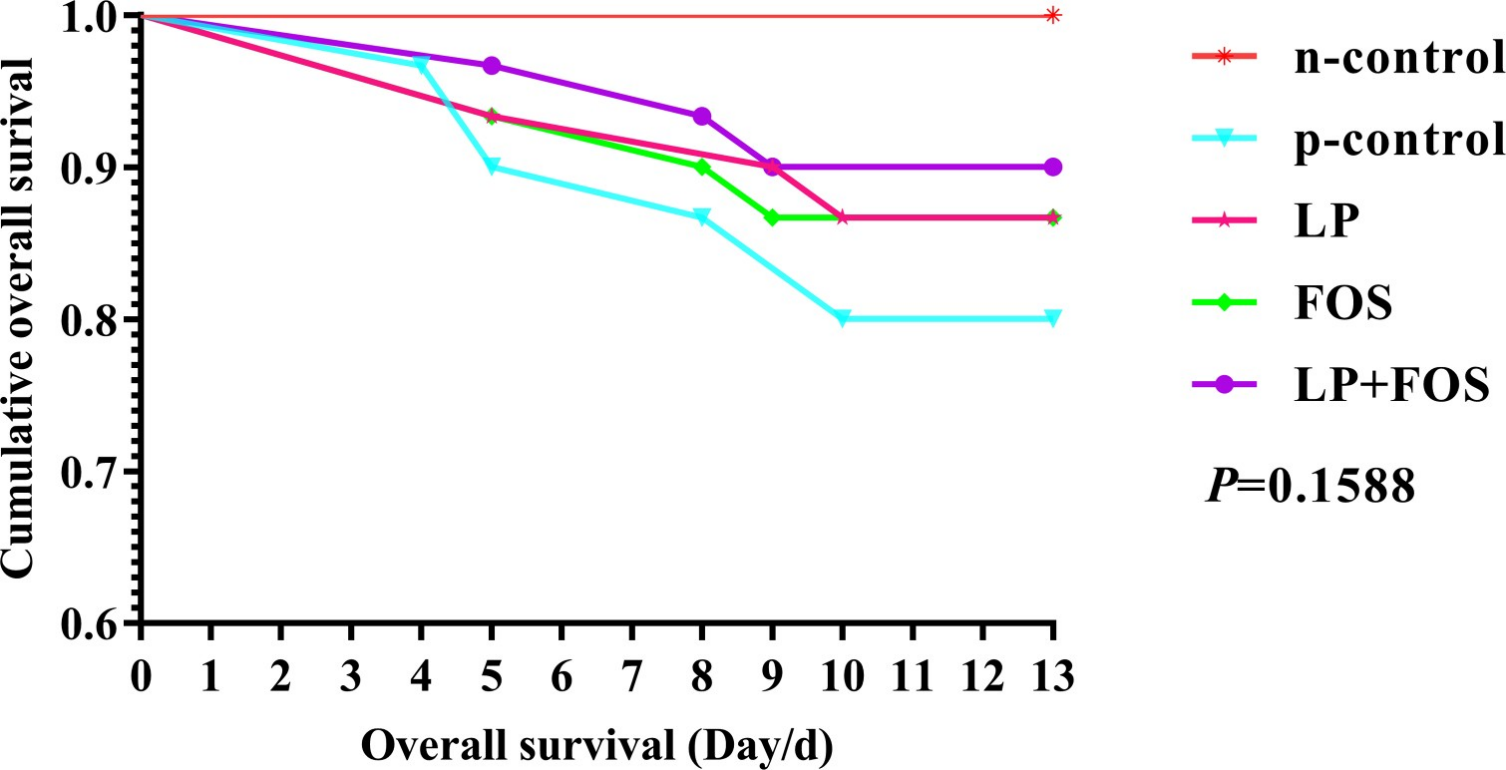
Kaplan–Meier curves showing survival of the broilers following *E*. *coli* O78 challenge for 13 days, *n* = 30.

The growth performance of the broilers is shown in Table 2. Challenge with *E*. *coli* O78 lowered the average weight at day 21 and the ADFI was decreased (*p*<0.001). Moreover, the ADG of broiler of FOS group showed no difference compared with n-control in 14–21 days and the whole period, which may demonstrated that FOS could alleviate the negative effect of *E*. *coli* O78 on the ADG to some extent. Moreover, FOS improved the bursal index compared with the p-control (*p*<0.05) and no difference was observed relative to the n-control. These data demonstrate that challenge with *E*. *coli* O78 caused the broilers to lose weight and damaged the bursa of Fabricius. Furthermore, Kaplan–Meier curves were plotted to examine the survival of the broilers during the period of challenging with *E*. *coli* O78; the results demonstrated that *L*. *plantarum* 15–1 and the combination of *L*. *plantarum* 15–1 and FOS reduced the mortality relative to the p-control group but did not result in zero mortality as with the n-control group (Fig 1).

**Table 2:** Influence of dietary supplementation on performance and mortality of broilers.

Parameter		Treatment (*E*. *coli* O_78_ challenge)				SEM	*P*-value
	n-control	p-control	LP	FOS	LP+FOS		
Average weight (kg)							
14 days	0.32	0.30	0.30	0.30	0.30	0.01	0.65
21 days	0.78^a^	0.59^b^	0.61^b^	0.68^a^^b^	0.59^b^	0.05	0.03
ADG (g/d)							
1–14 days	23.75	22.37	22.30	21.99	22.12	0.96	0.70
14–21 days	91.65^a^	68.32^b^	70.80^b^	79.20^a^^b^	67.28^b^	5.61	0.03
1–21 days	49.62^a^	39.87^b^	40.78^b^	43.79^a^^b^	39.32^b^	2.58	0.05
ADFI (g/d)							
1–14 days	30.87	28.57	28.00	27.76	28.79	0.99	0.99
14–21 days	102.92^A^	56.43^B^	61.20^B^	66.20^B^	58.77^B^	6.84	0.0003
1–21 days	95.94^A^	63.79^B^	67.14^B^	74.2^B^	64.04^B^	5.73	0.003
Bursal index (g.kg^-1^)							
14 days	2.86	2.41	1.98	2.02	2.28	0.27	0.25
21 days	2.40^a^	1.51^c^	1.76^b^^c^	2.32^a^^b^	2.07^a^^b^^c^	0.20	0.20
Mortality (%)							
1–21 days	0.00	13.33	10.00	13.33	10.00	NA	NA

ADFI: average daily feed intake. ADG: average daily gain. NA: not available. Mortality: mortality after challenging with *E*. *coli* O78.

^a, b, c^ means *p<*0.05, *n* = 6.

^A, B^ means *p<*0.001, n-control (broilers fed with basal diet and orally administered sterile saline); p-control (broilers fed with basal diet and orally administered *E*. *coli* O78); LP (broilers fed with basal diet supplemented with 1×10^8^ CFU/kg *L*. *plantarum* 15–1 and orally administered *E*. *coli* O78); FOS (broilers fed with basal diet supplemented with 5 g/kg FOS and orally administered *E*. *coli* O78); LP+FOS (broilers fed with basal diet supplemented with 1×10^8^ CFU/kg *L*. *plantarum* 15–1 and 5 g/kg FOS and orally administered *E*. *coli* O78).

### Jejunal morphology

**Fig 2:**
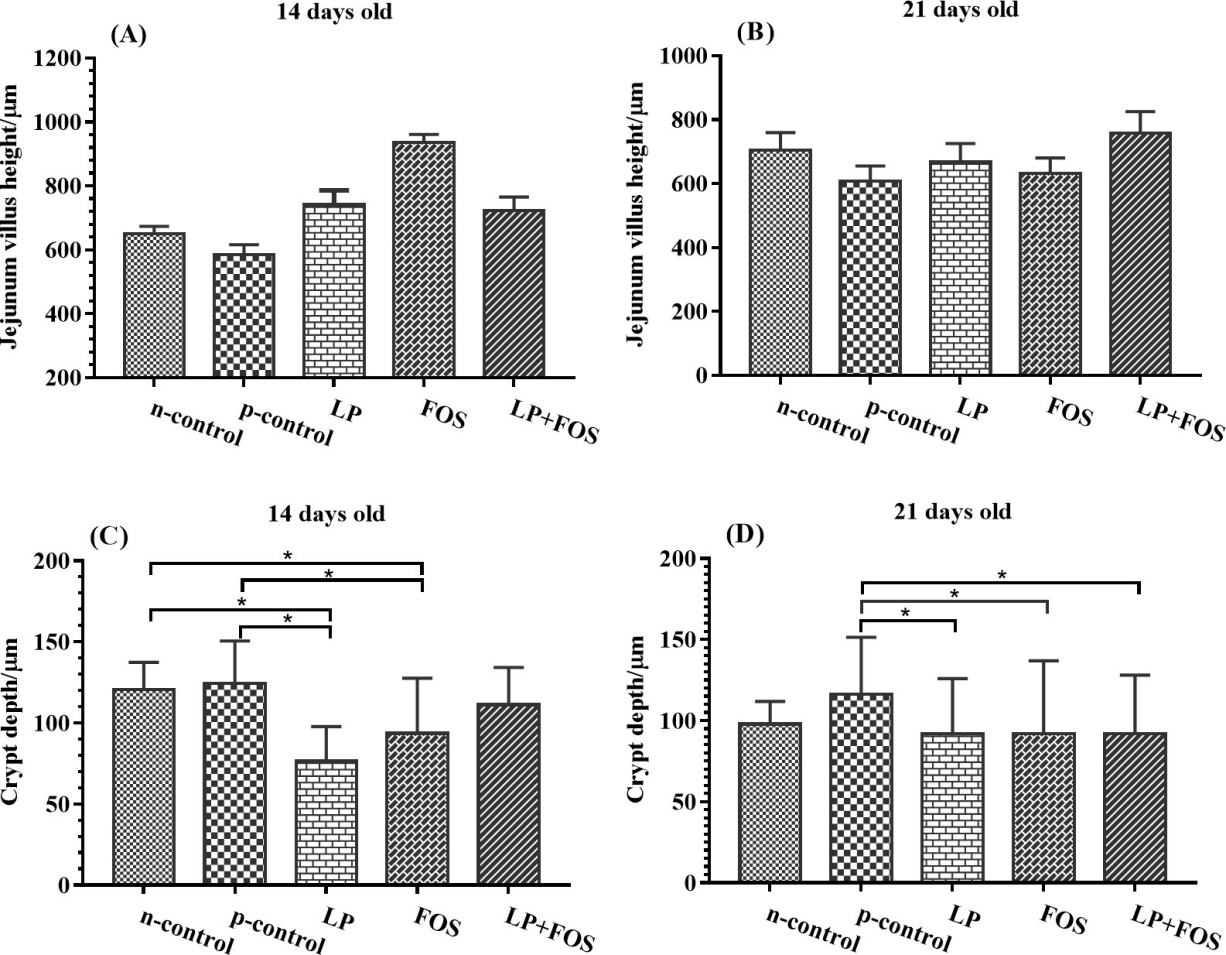
Influence of dietary supplementation on jejunal morphology of broilers after *E*. *coli* O78 challenge. (A) The villus height of broiler in 14 days old. (B) The villus height of broiler in 14 days old. (C) The crypt depth of broiler in 14 days old. (D) The crypt depth of broiler in 21 days old. * indicates *p*<0.05.

Supplementation with *L*. *plantarum* 15–1 or FOS decreased the crypt depth at day 14 relative to the n-control and p-control groups (Fig 2C, *p<*0.05). At day 21, a reduced crypt depth displayed in LP, FOS and LP+FOS group in comparison with the p-control group (*p<*0.05) but exhibited no difference relative to the n-control group (Fig 2D). No other significant differences were observed.

### Immune responses

**Fig 3:**
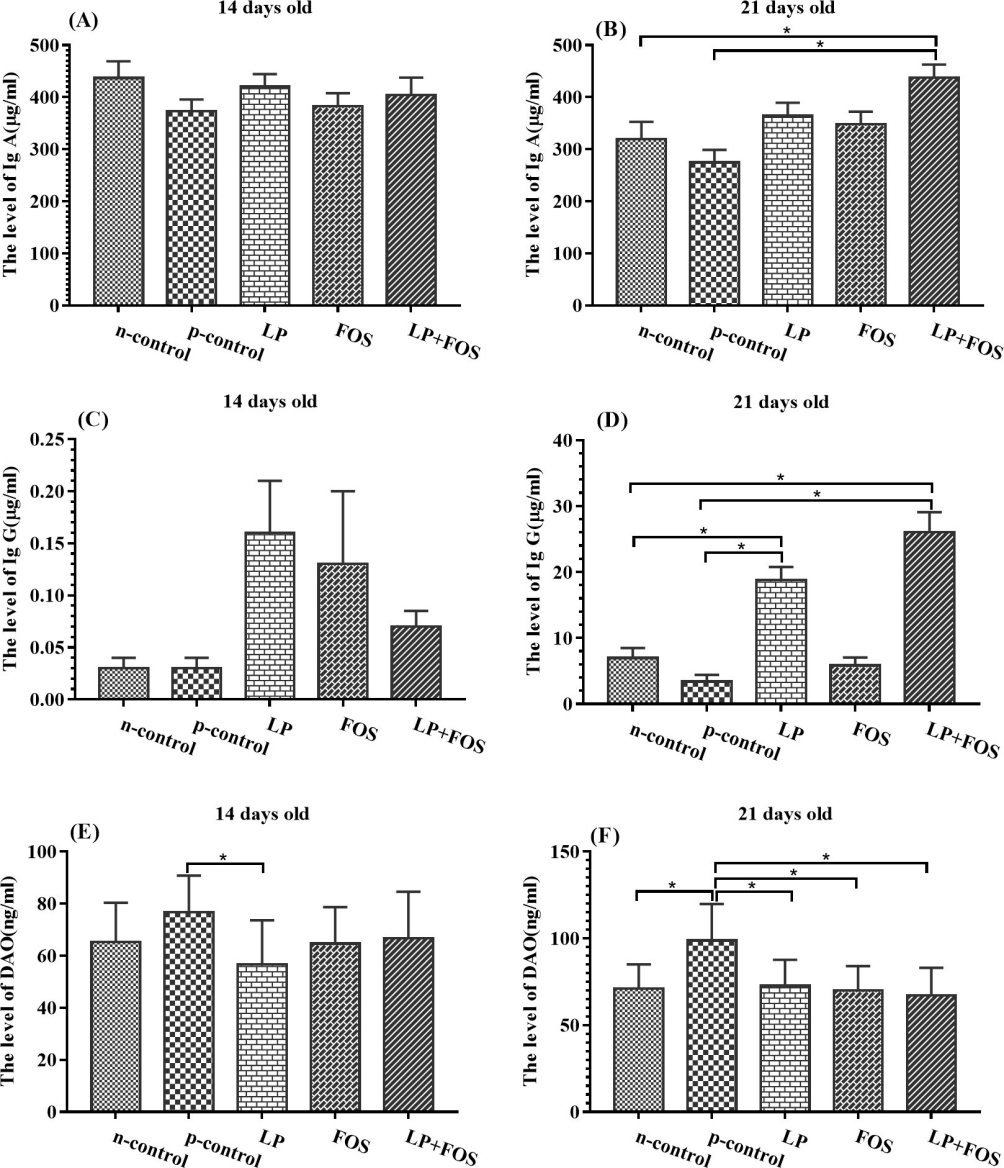
Influence of dietary supplementation on serum concentrations of IgA, IgG, and DAO in broilers. (A) The level of IgA in 14 days old. (B) The level of IgA in 21 days old. (C) The level of IgG in 14 days old. (D) The level of IgG in 21 days old. (E) The level of DAO in 14 days old. (F) The level of DAO in 21 days old. * indicates *p*<0.05.

The effects of *L*. *plantarum* 15–1 and FOS on the sero-immunity levels are presented in Fig 3. The level of DAO was reduced in LP group compared with the p-control group, whereas the other groups exhibited no differences at day 14 (Fig 3E, *p<*0.005). Moreover, at 21 day, the level of IgA and IgG was increased in LP+FOS group in relative with n-control and p-control (Fig 3B, *p*<0.005), and IgG was reduced in LP compared with n-control and p-control (Fig 3D, *p*<0.005). In addition, the level of DAO was decreased in p-control in relative with other four groups at day 21 (Fig 3F, *p*<0.005).

### Cecal short-chain fatty acids

**Fig 4:**
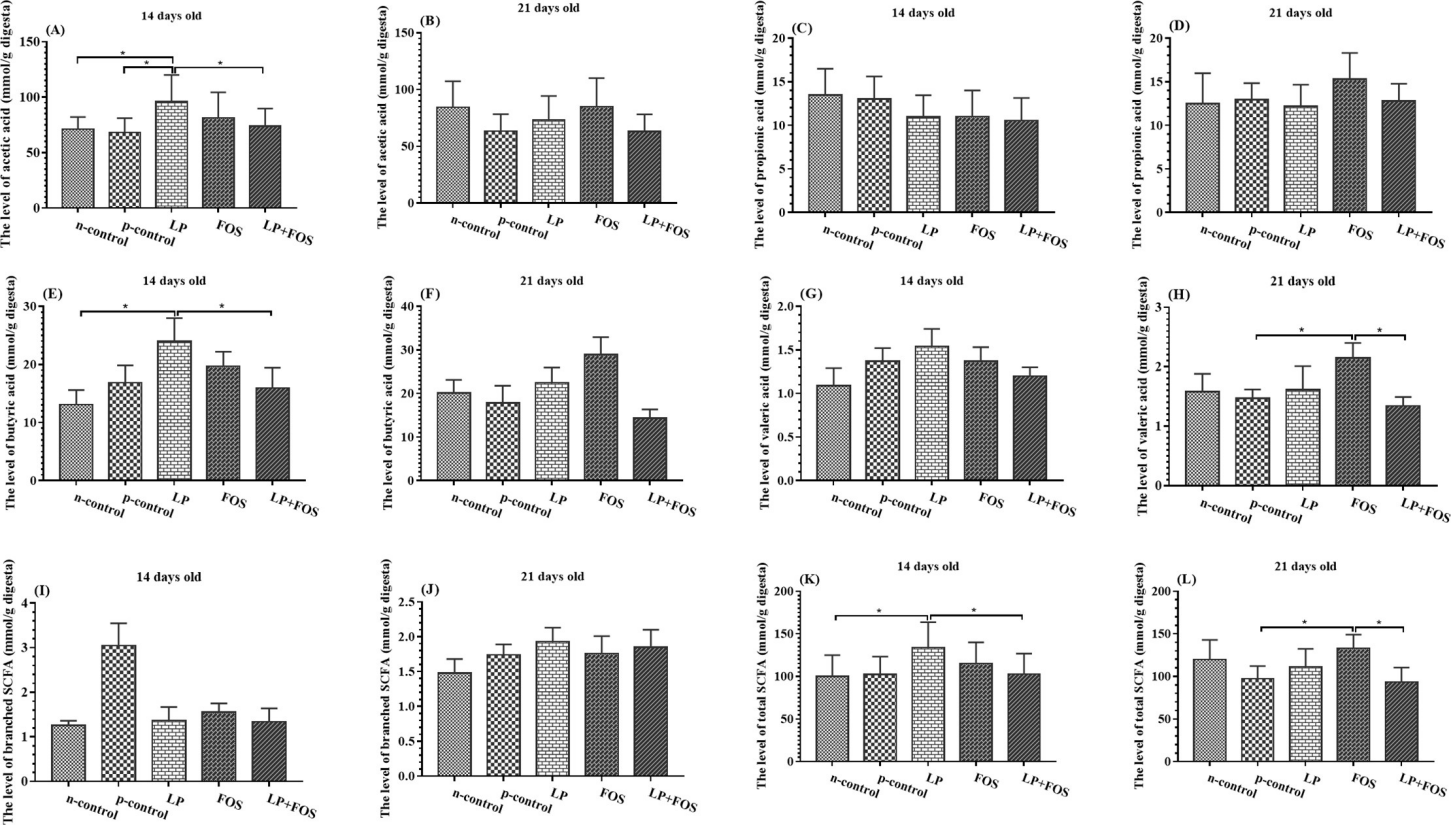
Influence of dietary supplementation on caecal SCFA levels of broilers at 14 and 21 days of age. (A) The level of acetic acid in 14 days old. (B) The level of acetic acid in 21 days old. (C) The level of propionic acid in 14 days old. (D) The level of propionic acid in 21 days old. (E) The level of butyric acid in 14 days old. (F) The level of butyric acid in 21 days old. (G) The level of valeric acid in 14 days old. (H) The level of valeric acid in 21 days old. (I) The level of branched SCFA in 14 days old. (J) The level of branched SCFA in 21 days old. (K) The level of total SCFA in 14 days old. (L) The level of total SCFA in 21 days old. * indicates *p*<0.05.

The cecal SCFA content was analyzed using gas chromatography after thawing the samples at 4°C. The results demonstrated that the level of caecal acetic acid/butyric acid (Fig 4A and 4E) was increased in LP group in relative to n-control and p-control/n-control respectively at 14 days old (*p*<0.05). Moreover, the concentration of valeric acid and the total SCFA was increased in FOS group in comparison with p-control at 21 days old (Fig 4H and 4L, *p*<0.05).

## Discussion

Reducing the immune and intestinal damage caused by pathogenic *E*. *coli* is of great significance for improving poultry health. FOS are conducive to animal growth and provide resistance to pathogenic bacteria. FOS are a preferred substrate for improving the growth of *Bifidobacteria* and promoting its association with the mucous membranes of the host, which hinders the binding of pathogenic bacteria to the intestinal tract [24]. The current results suggest that *L*. *plantarum* 15–1 and FOS can ameliorate the intestinal damage induced by *E*. *coli* O78 and increase the immune response, such as by increasing the levels of IgA and IgG and reducing the serum concentration of DAO. Moreover, *L*. *plantarum* 15–1 and FOS increased the SCFA levels in the cecal contents, which may help resist the invasion of pathogenic bacteria. These results indicate that *L*. *plantarum* 15–1 and FOS could help maintain the health status of broilers.

*E*. *coli* is associated with the deterioration of animal health, including weight loss, diarrhea, mortality, and necrotizing enteritis [25]. Research by Porcheron has demonstrated that FOS may be capable of regulating an extra-intestinal pathogenic *E*. *coli* strain, and this property is associated with a gene cluster called the *fos* locus, which plays a major role in intestinal colonization [26]. These results support the idea that the probiotic bacteria of the microbiota can metabolize in the intestine and decrease the population of pathogenic bacteria. However, no significant differences were observed in terms of the growth performance. The same findings have also been reported in other studies; dietary supplementation with 0.5% FOS did not influence on the growth performance of broilers [18]. In contrast, some studies have reported that FOS enhanced the feed efficiency (FCR) but reduced the feed intake and daily live weight gain in the absence of challenge with *E*. *coli* [27]. G.-B. Kim et al. reported that supplementation with 0.25% FOS improved the growth performance of broilers at 28 days [18]. Furthermore, Xu et al. found that FOS increased the body weight and feed conversion ratio, and this effect was considerably greater in three-week-old animals than in one-week-old animals [28]. These findings may indicate that it takes time for the beneficial microorganisms to utilize the FOS and become the dominant microflora to maintain the balance of the intestinal microbial environment and improve the growth of broilers challenged with *E*. *coli*. The results of the present study were in agreement with those of Xu et al. Numerous bacteria have been used as probiotics, including *Lactobacillus*, *Leuconostoc*, *Pediococcus*, *Bifidobacterium* and *Enterococcus*, which promote the growth of animals [29, 30]. Peng et al. reported that the use of *Lactobacillus plantarum* B1 led to higher ADG during the finisher period [14]. Saffar et al. examined the use of probiotics to reduce ascites in broilers in high-altitude areas, and the results revealed that probiotics reduce the ascites mortality has a role in promoting, but not to compensate for the growth of a strong impact [31]. This study indicated that *L*. *plantarum* and FOS improved the performance of broilers and reduced the mortality after challenging with *E*. *coli* O78. Moreover, *L*. *plantarum* 15–1 and FOS treatment decreased the crypt depth at days 14 and 21. However, there are no differences in body weight compared with the p-control group.

In poultry, a healthy intestinal tract is essential for absorbing nutrients and providing a barrier against pathogenic bacteria. Awad et al. reported that *Lactobacilli* exert a positive effect on the gastrointestinal tract as they increase feed consumption and nutrient absorption from the intestines [32]. The intestines are an important site of nutrient absorption, and the absorption efficiency is associated with the surface area due to an increasing villus and mucosa thickness [33]. Saffar et al. examined the use of probiotics to reduce ascites in broilers in high-altitude areas, and the results revealed that probiotics reduce the ascites mortality has a role in promoting, but not to compensate for the growth of a strong impact [34]. Pan et al. reported that challenge with enterotoxigenic *E*. *coli* K88 led to atrophy of the villi and destruction of the intestinal morphology, whereas supplementation with the probiotic (*Bacillus licheniformis* and *Saccharomyces cerevisiae*) protected the intestinal barrier function from the *E*. *coli* K88, indicating that probiotics could potentially replace antibiotics in the treatment of diarrhea [35]. Awad et al. reported that dietary supplementation with *Lactobacillus* increased the villus height and villus height:crypt depth ratio in broilers [36], and Song et al. reported that supplementation with a mixed probiotic consisting of *Lactobacillus plantarum*, *Bacillus licheniformis*, and *Bacillus subtilis* increased the jejunal villus height and decreased small intestinal coliforms [37]. The present study demonstrates that the addition of *L*. *plantarum* 15–1 to a basal diet improves the jejunal tissue morphology.

The serum level of DAO reflects the structure, function, and integrity of the small intestinal mucosa. Under normal circumstances, the serum level of DAO is very low, but it increases after damage to the intestinal mucosa [38]. Several studies have demonstrated that the consumption of probiotics and prebiotics improves host immunity by increasing the concentration of IgA. C.H. Kim et al. found that dietary supplementation with FOS increased the IgA levels in laying hens [39]. In contrast, G.-B. Kim et al. reported no difference in the plasma concentrations of IgA and IgG between broilers fed a diet supplemented with FOS and other treatment groups [18]. Maragkoudakis et al. also reported no differences in the plasmas levels of IgA, IgM, and IgG in goats upon dietary supplementation with FOS [40], and Gürbüz et al. reported no differences in the concentrations of IgA, IgM, and IgG in horses [41]. In this study, dietary supplementation with *L*. *plantarum* 15–1, FOS, and their combination increased the serum levels of IgA and IgG at day 21 and decreased the DAO levels.

SCFAs are the pivotal metabolite during the microbial fermentation of indigestible carbohydrates in the large intestine and are used by the colonic mucosa to enhance the epithelial barrier [14, 42]. Lactic acid bacteria enhance the physical barrier of the host intestine by increasing the concentration of SCFAs, as high levels of SCFAs inhibit the growth and reproduction of pathogenic bacteria. SCFAs are the final products of microbial fermentation and are absorbed through the colonic mucosa [43]. SCFAs are ideal biorenewable chemicals for inhibiting *E*. *coli* that function by chemically damaging the cell membrane of the pathogen [44]. The application of *Lactobacillus reuteri* DPC16 enhances the level of SCFAs, thus increasing the intestinal acidity, which is conducive to reducing the pathogen population in the gut of chickens [45]. Butyrate is also a direct source of energy for the colonic epithelium, possesses anti-inflammatory properties, and can enhance the colonic defense barrier [46]. Peng et al. reported that *L*. *plantarum* B1 increased the SCFA levels in the cecum [47]. In this study, dietary supplementation with *L*. *plantarum* 15–1, FOS, or both led to increased levels of total SCFAs, especially acetic acid and butyric acid at day 14 and valeric acid at day 21. This may partially account for the mortality reduction upon administration of probiotics. However, it is notable that one study reported that synbiotics containing two or more substances did not induce an additive effect with respect to growth performance, intestinal microbial population, or SCFA levels [48]. This discrepancy may originate from the different properties of prebiotics and probiotics.

In conclusion, dietary supplementation with FOS and *L*. *plantarum* 15–1 improved the intestinal morphology, enhanced the immune response, and increased the SCFA concentration in the cecum in broilers challenged with *E*. *coli* O78. Moreover, supplementation with *L*. *plantarum* 15–1 and FOS had no effect on growth performance but decreased the mortality of the challenged broilers. These results indicate that dietary supplementation with FOS and *L*. *plantarum* 15–1 may ameliorate the negative effects of *E*. *coli* O78.

## Supporting information

S1 Dataset Minimal data set. (XLSX)
