# Correction: Effects of *Lactobacillus plantarum* 15-1 and fructooligosaccharides on the response of broilers to pathogenic *Escherichia coli* O78 challenge

**DOI:** 10.1371/journal.pone.0222877

**Published:** 2019-09-17

**Authors:** Sujuan Ding, Yongwei Wang, Wenxin Yan, Aike Li, Hongmei Jiang, Jun Fang

Reference 25 is incorrect. The correct reference is: Casewell M, Friis C, Marco E, McMullin P, Phillips I. The European ban on growth-promoting antibiotics and emerging consequences for human and animal health. The Journal of antimicrobial chemotherapy. 2003;52(2):159–61. Epub 2003/07/03. doi: 10.1093/jac/dkg313. PubMed PMID: 12837737.
